# Genetic and genomic analysis of classic aniridia in Saudi Arabia

**Published:** 2011-03-11

**Authors:** Arif O. Khan, Mohammed A. Aldahmesh, Fowzan S. Alkuraya

**Affiliations:** 1 Division of Pediatric Ophthalmology, King Khaled Eye Specialist Hospital, Riyadh, Saudi Arabia; 2 Department of Genetics, King Faisal Specialist Hospital and Research Center, Riyadh, Saudi Arabia; 3 Department of Pediatrics, King Khalid University Hospital and College of Medicine, King Saud University, Riyadh, Saudi Arabia; 4 Department of Anatomy and Cell Biology, College of Medicine, Alfaisal University, Riyadh, Saudi Arabia

## Abstract

**Purpose:**

To determine the genetic and genomic alterations underlying classic aniridia in Saudi Arabia, a region with social preference for consanguineous marriage.

**Methods:**

Prospective study of consecutive patients referred to a pediatric ophthalmologist in Saudi Arabia (2005–2009). All patients had paired box gene 6 (*PAX6*) analysis (sequencing and multiplex ligation-dependent probe amplification analysis if sequencing was normal). If *PAX6* analysis was negative, the following were performed: candidate gene sequencing (forkhead box C1 [*FOXC1*], paired-like homeodomain transcription factor 2 [*PITX2*], cytochrome P450, family 1, subfamily B [*CYP1B1*], paired-like homeodomain transcription factor 3 [*PITX3*], and v-maf avian musculoaponeurotic fibrosarcoma oncogene homolog [*MAF*]) and molecular karyotyping by array competitive genomic hybridization (250K single nucleotide polymorphism (SNP) arrays).

**Results:**

All 12 probands (4 months – 25 years of age; four boys and eight girls) had lens opacity and foveal hypoplasia in addition to no  grossly visible iris. Four cases were familial. All cases were products of consanguineous unions except for three, one of which was endogamous. Heterozygous *PAX6* mutations (including two novel mutations) were detectable in all but two cases, both of which were sporadic. In one of these two cases, the phenotype segregated with homozygosity for a previously-reported pathogenic missense *FOXC1* variant (p.P297S) when homozygosity for chromosome 11q24.2 deletion (chr11:125,001,547–125,215,177 [rs114259885; rs112291840]) was also present. In the other, no genetic or genomic abnormalities were found.

**Conclusions:**

The classic aniridia phenotype in Saudi Arabia is typically caused by heterozygous *PAX6* mutations, even in the setting of enhanced homozygosity from recent shared parental ancestry. For *PAX6*-negative cases, interaction between missense variation in an anterior segment developmental gene and copy number variation elsewhere in the genome may be a potential mechanism for the phenotype.

## Introduction

Classic aniridia (OMIM 106210) is a panocular disorder which, in addition to lack of grossly visible iris, is characterized by keratopathy, lens opacity, juvenile-onset glaucoma, foveal hypoplasia, and optic nerve hypoplasia [1]. This classic phenotype is caused by heterozygous mutation in the ocular developmental gene paired box gene 6 (*PAX6*; OMIM *607108; 11p13), typically through haploinsufficiency [2]. For cases in which the contiguous gene Wilms tumor 1 (*WT1*; OMIM 194070; 11p13) is also disrupted, affected children are typically syndromic and at risk for juvenile Wilms tumor of the kidney (WAGR syndrome, OMIM 194072) [3]. Previous studies have shown that dominant *PAX6* mutations are the only known cause of classic aniridia. However, even with comprehensive analysis of *PAX6* including copy number analysis, up to 10% of classic anirida cases have no identifiable mutation [2,4].

Congenital iris abnormality in general can also be due to heterozygous *PAX6* mutation but is both phenotypically and genotypically more heterogenous. Heterozygous mutation in the ocular developmental gene forkhead box C1 (*FOXC1*; OMIM *601090; 6p25) or paired-like homeodomain transcription factor 2 (*PITX2*; OMIM *601542; 4q25-q26) causes the Axenfeld-Rieger spectrum (OMIM 602482, 180500, 137600), an anterior segment dysgenesis which can include mild iris stromal hypoplasia, corectopia, polycoria, iris pigment border hyperplasia (“ectropion uvea”), and/or virtually complete lack of iris [5]. *FOXC1* duplication can cause the phenotype as well [6,7]. Affected children have a propensity for congenital or juvenile glaucoma, can have neural crest-related non-ocular findings (e.g., maxillary hypoplasia, dental anomalies), and can show significant intrafamilial phenotypic variation for the same mutation [5,8,9]. Congenital iris abnormality can also occur in the setting of congenital glaucoma from homozygous or compound heterozygous mutations in cytochrome P450, family 1, subfamily B (*CYP1B1*; OMIM *601771; 2p22-p21) [10], in the setting of anterior segment mesenchymal dysgenesis due to heterozygous mutation in paired-like homeodomain transcription factor 3 (*PITX3*; OMIM +602669; 10q25) [11], and in the setting of anterior segment dysgenesis due to heterozygous mutation in v-maf avian musculoaponeurotic fibrosarcoma oncogene homolog (*MAF*; OMIM *177075; 16q22-q23) [12]. The variation in iris phenotype for a given mutation that has been observed in *FOXC1*-related disease as well as in other genetic causes of congenital iris abnormality is likely related to the influence of yet unidentified modifiers.

Although a small percentage of aniridia cases have no detectable *PAX6* mutation, to the best of our knowledge no other genotype has been associated with the classic aniridia phenotype. We propose two potential causes for *PAX6*-negative classic anirida: 1) genomic alteration that affect cis or trans regulatory elements of *PAX6*, or 2) point mutations or deletions in other genes known to be involved in the pathogenesis of congenital iris abnormalities. Genome-wide copy number analysis of classic aniridia cases with negative *PAX6* sequencing can investigate the first possibility. In addition to interrogation for genomic alteration in a genome-wide fashion, this would also further characterize *PAX6* deletions. Regarding the second possibility, i.e., mutations in other known genes, gross deletions would also be uncovered by genome-wide copy number analysis while sequencing variants can be investigated by direct sequencing of a panel of genes known to cause congenital iris abnormalities when mutated. Moreover, if recessive mutation in other known genes can cause classic aniridia, this would most likely be uncovered by the study of affected consanguineous families, for whom a recessive cause is more likely to be found if a recessive cause for the phenotype exists [13]. In the current study, we analyze the underlying genotype of classic aniridia in mostly consanguineous patients by *PAX6* analysis and, for *PAX6*-negative cases, by candidate gene sequencing and array genomic copy number variation analysis.

## Methods

Institutional review board approval was obtained for this study (KFSHRC IRB #2070023

KKESH IRB #?????). Consecutive patients with classic aniridia referred to the pediatric ophthalmology service of one of the authors (A.O.K.) from 2005 to 2009 were prospectively enrolled in the study. Classic anirida was defined as no grossly visible iris in addition to keratopathy, lens opacity, and/or foveal hypoplasia [1]. Patients underwent complete ophthalmic examination and venous blood sampling for analysis. The strategy for genetic and genomic analysis was as follows: If direct sequencing did not reveal *PAX6* mutation, multiplex ligation-dependent probe amplification (MLPA) was performed to assess for *PAX6* deletions. If this was normal, the candidate genes *FOXC1, PITX2*, *CYP1B1, PITX3*, and *MAF* were sequenced. In addition, genomic molecular karyotyping was performed via array-based comparative genomic hybridization (array CGH) in all cases that lacked an identifiable *PAX6* mutation. When available and appropriate, relatives underwent ophthalmic examination and venous blood sampling for confirmatory genetic analysis.

### DNA extraction

Genomic DNA was extracted from whole blood anti-coagulated with EDTA using the Purgene Gentra DNA Extraction Kit (Cat. # D-5000; Gentra Systems, Minneapolis, MN) according to the manufacturer’s instructions. The DNA was quantified spectrophotometrically and stored in aliquots at −20 °C until required.

### PCR amplification and DNA sequencing

PCR amplification was performed on a thermocycler (DNA Engine Tetrad, MJResearch, Inc., Hercules, CA) in a total volume of 25 µl, containing 10 ng DNA, 50 mM KCl, 10 mM Tris-HCl (pH 9.0), 1.5 mM MgCl_2_, 0.1% Triton X-100, 0.25m M of each dNTP, 0.8 µM of each primer and 0.5 Units of Taq polymerase (D-40724; QIAGEN, Hilden, Germany). For PCR, an initial denaturation step at 95 °C for 10 min was followed by 40 cycles of denaturation at 95 °C for 30 s, annealing at 59 °C for 30 s and extension at 72 °C for 30 s followed by a final extension step of 72 °C for 10 min. Genomic DNA of each patient was screened for coding regions and boundary site variants by sequencing reactions using BigDye® Terminator v 3.0 (Applied Biosystems, Inc., Foster City, CA). Following the manufacturer’s instructions, sequencing reactions were desalted and unincorporated nucleotides removed using ethanol precipitation and re-suspended in a deionized distilled water for injection on a Applied Biosystems 3730xl DNA Analyzer (Applied Biosystems, Inc.). Sequence analysis was performed using the SeqManII module of the Lasergene (DNA Star Inc., Madison, WI) software package and was compared to the relevant reference sequence.

### MLPA analysis

MLPA analysis of *PAX6* was performed using the commercial Kit P219 PAX6 (MCR Holland, Amsterdam, The Netherlands). The manufacturer’s instructions were followed and data was analyzed using Coffalyser software (MCR Holland).

### Molecular karyotyping

Affymetrix Genechip Human Mapping 250K SNP arrays (Affymetrix, Santa Clara, CA) were used. All procedures were performed according to Affymetrix standard protocols. The SNP call rate and sample mismatch report was determined with GTYPE software (Affymetrix). Two algorithms, the Affymetrix® Genotyping Console™ and Copy Number Analyzer for GeneChip® arrays (CNAG) Version 3.021 software were used to infer copy number variation among affected individuals based on the hybridization intensity signal of the probes.

## Results

**Figure 1:**
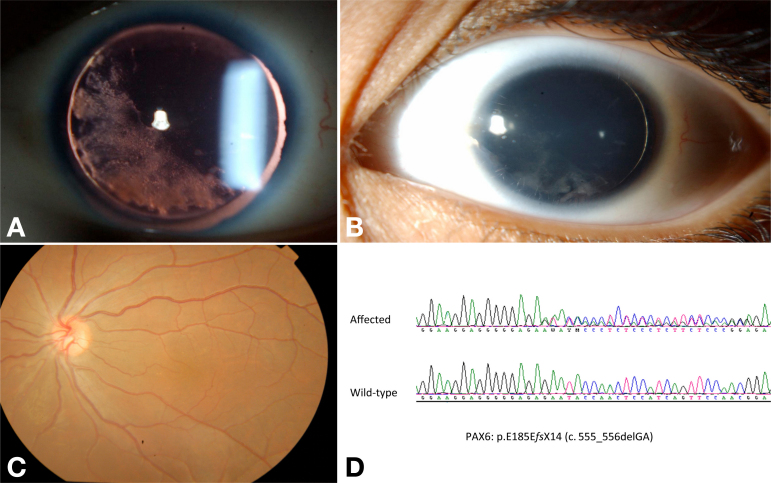
Patient 2, without pharmacologic mydriasis. **A**: Retroillumination shows lack of iris and the lenticular changes (left eye shown). **B**: Diffuse illumination reveals surface keratopathy (left eye shown). **C**: There was no defined fovea by indirect ophthalmoscopy (left eye shown). **D**: sequencing revealed a novel *PAX6* mutation (p.E185EfsX14).

**Figure 2:**
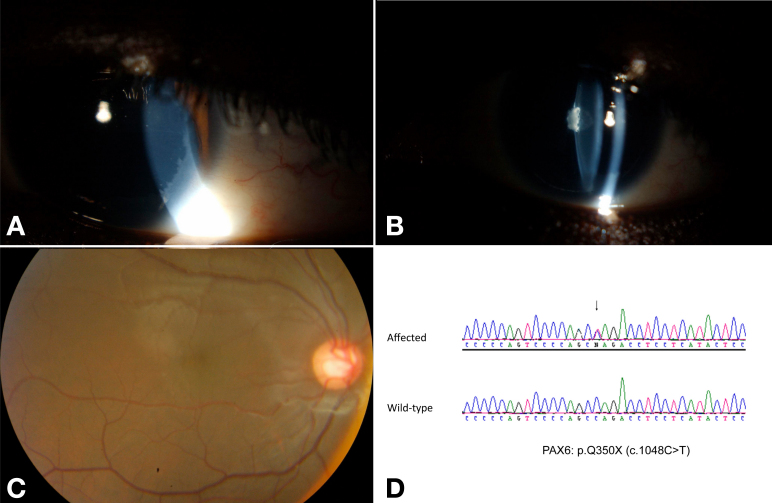
Patient 6, without pharmacologic mydriasis. **A**: Slit illumination revealed surface limbal keratopathy (right eye shown). **B**: Slit illumination shows lack of iris and a posterior lenticular opacity (right eye shown). **C**: The fovea is not well defined (right eye shown). **D**: sequencing revealed a novel *PAX6* mutation (p.Q350X).

Twelve probands (four familial, eight sporadic) were included in the study – four boys and eight girls who ranged from 4 months to 25 years of age. Two of the familial cases were previously-reported [14]. All patients were products of consanguineous unions except for three cases, one of which was endogamous. None had associated non-ocular congenital malformation or complicated birth history and all had had comprehensive physical examination by a pediatrician. All probands had lens opacity, foveal hypoplasia, and gross lack of iris; in addition, all probands had keratopathy except for two sporadic cases (patients 7 and 12 in Table 1). All four familial cases and six of the eight sporadic cases had *PAX6* mutation, including two novel *PAX6* mutations (Table 1, Figure 1, and Figure 2). The two sporadic cases without detectable *PAX6* mutation (patients 7 and 9 in Table 1), both from consanguineous families, are discussed further below.

**Table 1:** Summary of the results of molecular analysis of patients with classic aniridia.

**ID**	**Inbred?**	**Age**	**Sex**	**#**	**PAX6 mutation**	**Gene analysis**	**Copy number variation**	**Comment**
1	Consang	6	F	9	p.Arg240X (c.1195C>T)	N/A	N/A	Family from reference [14]
2	No	10	F	4	**p.E185EfsX14 (c.555_556delGA)	N/A	N/A	
3	Consang	9	F	3	p.Pro39ArgfsX14 (c.112del1)	N/A	N/A	
4	Consang	8	F	4	p.Asn273IlefsX91 (c.delA1294)	N/A	N/A	Family from reference [14]
5	Consang	25	M	1	p.Arg240X (c.718C>T)	N/A	N/A	
6	Consang	9	M	1	** p.Gln350X (c.1048C>T)	N/A	N/A	developed juvenile glaucoma
7	Consang	8	F	1	none	no mutation found	none found	no keratopathy
8	No	7	M	1	p.Ala37ProfsX16 (c.109del1)	N/A	N/A	accommodative esotropia
9	Consang	7	F	1	none	homozygous p.Pro297Ser FOXC1 (c.889C>Tr)	chr11q24.2:125,001,547-125,215,177 (rs114259885;rs112291840)	
10	Consang	3	M	1	p.Ser167X (c.500C>A)	N/A	N/A	optic nerve hypoplasia
11	Consang	1	F	1	*PAX6* gene deletion (see copy number variation column)	N/A	chr11:30,877,006-32,440,841 (1,563,836 bp)	
12	Endogom	4/12	F	1	*PAX6* and *WT*1 gene deletion (see copy number variation column)	N/A	chr11:27,206,264-42,280,976 (15,074,713 bp)	Non-consanguineous but endogamous; no keratopathy

#denotes number of affected individuals), N/A: not applicable; **denotes novel mutation. Consang: consanguineous; Endogom: endogamous.

**Figure 3:**
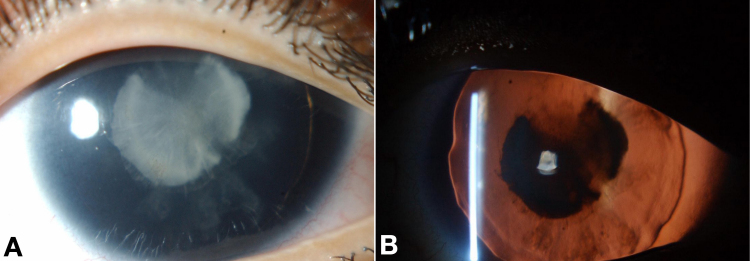
Patient 7, without pharmacologic mydriasis. **A**, **B**: In addition to lack of iris, this patient without detectable *PAX6* mutation had lenticular opacity and foveal hypoplasia (not shown). Further genetic and genomic analyses were unremarkable. The right eye is shown.

Patient 7 (Figure 3) had classic aniridia in that in addition to lack of iris she had lens opacity and foveal hypoplasia. She had normal genetic and genomic analysis; no underlying cause was found for the ocular phenotype.

**Figure 4:**
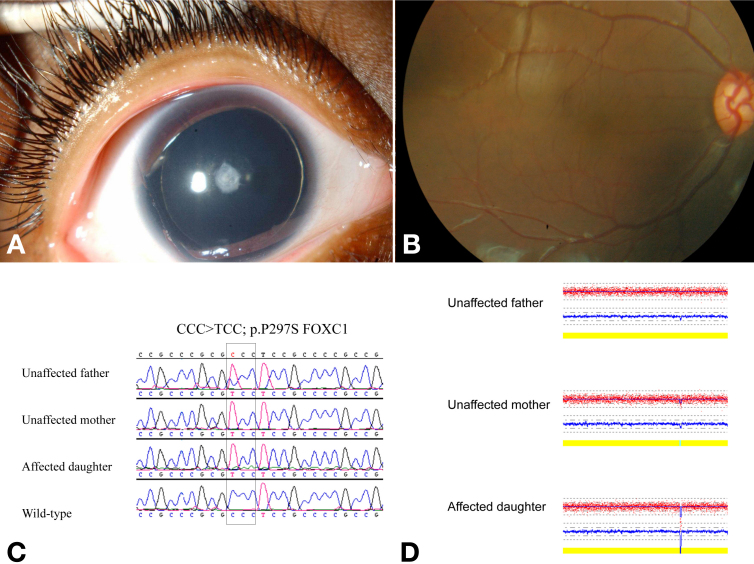
Patient 9, without pharmacologic mydriasis. **A**, **B**: In addition to lack of iris, this patient without detectable *PAX6* mutation had anterior lens opacity, limbal keratopathy (not shown), and foveal hypoplasia. **C**: Further genetic and genomic analyses revealed homozygosity for both a previously-described heterozygous *FOXC1* mutation (p.P279S) and a for chromosome 11q24.2 deletion (**D**) while neither unaffected parent was homozygous for both.

Patient 9 (Figure 4) had classic aniridia in that in addition to lack of iris she had keratopathy, lens opacity, and foveal hypoplasia. She was homozygous for a missense variant in *FOXC1* (p.P297S) that was previously been reported as pathogenic and as a cause for dominant anterior segment dysgenesis in two unrelated individuals [15]. However, her father was heterozygous for the variant while her mother was homozygous and neither parent had significant ophthalmic findings despite careful clinical ophthalmic examination with attention to the anterior segment and fovea. Molecular karyotyping revealed homozygosity (nullizygosity) for a chromosome 11q24.2 deletion (chr11:125,001,547–125,215,177 [rs114259885 and rs112291840]) present in the child and heterozygosity (hemizygosity) for the deletion in both parents. Thus the child's phenotype segregated with homozygosity for the *FOXC1* missense variant in conjunction with homozygosity for the deletion (Figure 4). The region of this deletion contains five genes (CHK1 checkpoint homolog [*CHEK1*], acrosomal vesicle protein 1 [*ACRV1*], prostate and testis expressed 4 [*PATE4*], prostate and testis expressed 2 [*C11ORF38*], and *FLJ41047*), none of which has known ocular function. Neither the FOXC1 variant nor the deletion was found in 100 ethnically-matched controls.

## Discussion

In this series of classic aniridia in mostly consanguineous families from Saudi Arabia, the heterozygous *PAX6* mutation was detected in 4/4 familial cases and 6/8 sporadic cases. One P*AX6*-negative case harbored homozygosity for both a previously-reported pathogenic missense *FOXC1* variant and for a deletion on chromosome 11q24.2; the unaffected parents were heterozygous or homozygous for the *FOXC1* variant and both were heterozygous for the deletion. The other P*AX6*-negative case remained idiopathic despite candidate gene sequencing and whole genome copy number analysis.

Studies of consanguineous families are more likely to uncover a recessive cause for a given phenotype if a recessive cause exists because of parental shared recent ancestry. Every individual is a heterozygous carrier for mutated alleles that would potentially cause recessive disease in the homozygous (or compound heterozygous) state. Consanguineous marriage increases the expression of rare recessive disease because unless carriers are related, they are unlikely to marry a partner who carries the same disorder [16]. In the current study of classic aniridia of mostly inbred families, 3/3 familial cases from consanguineous families and 6/8 sporadic cases from consanguineous or endogamous families harbored heterozgyous *PAX6* mutation. Thus even in the setting of enhanced homozygosity from recent shared parental ancestry, heterozgyous *PAX6* mutation typically underlies the phenotype of classic aniridia.

Two patients, both from consanguineous families, had no detectable *PAX6* mutation. Although no genetic or genomic cause for classic aniridia was found in one (Patient 7, Figure 3), in the other (Patient 9, Figure 4) there was a homozygous *FOXC1* missense variant previously reported as responsible for anterior segment dysgenesis in the heterozygous state [15]. However, both unaffected and otherwise normal parents of the patient harbored this variant – in the heterozygous state in the father and in the homozygous state in the mother – and both unaffected parents had no significant ophthalmic findings. Additional analysis by array CGH revealed a homozygous deletion in the child on chromosome 11q24.2 that was present in the heterozygous state in both parents. One can speculate that either the homozygous 11q24.2 deletion alone or in concert with the homozygous *FOXC1* missense variant was associated with the classic aniridia phenotype. The latter seems more plausible as the genes affected by the deletion do not have known ocular function while p.P297S FOXC1 has been previously described as pathogenic [15]. Functional work suggests that p.P297S FOXC1 alters interaction with other yet unidentified factors involved in FOXC1 transactivation and degradation, thus extending the half-life of the protein and causing an increased dosage effect akin to the mechanism of *FOXC1* duplication [15]. Consistent with this, cases associated with p.P297S FOXC1 as well as those associated with *FOXC1* duplication have been reported with phenotypes of iridogoniodysgeneis that can resemble the iris appearance of patients with classic anirida [15,17]. While the genes contained within this 11q24.2 deletion are not known to be involved in eye development, such a role has not been excluded. In addition, we cannot exclude the possibility that non-coding regulatory elements for *PAX6* may exist within this region. Either of these two hypothesized causal links could be either sufficient or dependent on the *FOXC1* mutation. Patient 9 also had developmental delay, which may or may not have been related to the deletion alone or in combination with p.P297S FOXC1.

For a particular mutation in a given gene, differences in a given individual's background genotype and environmental exposure cause intrafamilial phenotypic variability. Intrafamilial phenotypic variability has been well documented for *FOXC1*-related disease [5,9]. Background genomic copy number variation – both deletion and repeats – can increase susceptibility to ocular disease and contribute to phenotypic variability [18,19]. In patient 9 from this study (Figure 4), the homozygous deletion may have been responsible for the expression of a p.P279S FOXC1-related anterior segment phenotype, i.e., the homozygous deletion is a genomic explanation for the intrafamilial phenotypic variability and the observed classic aniridia in patient 9. Although there have been reports of lack of visible iris in patients with heterozygous *FOXC1* [20] as well as heterozygous *PITX2* [21] mutations, these previously-reported patients would not be considered classic aniridia as they did not have documented keratopathy, lens opacity, or foveal hypoplasia. In addition, the previously-reported patient [20] with lack of iris and heterozgyous *FOXC1* mutation also had obvious newborn glaucoma, which is not part of classic aniridia [10]. There is a previous report of gross lack of iris and concurrent keratopathy associated with heterozygous *FOXC1* mutation; this was in a baby boy who also had severe newborn glaucoma [22]. Again, his phenotype would not be considered classic aniridia because of newborn glaucoma [10].

Our finding of a homozygous deletion of a previously unreported copy number variation in a patient with this phenotype could be interpreted as a rare recessive form. However, this case has to be viewed in the context of the *FOXC1* mutation and lack of other siblings in whom we can verify the segregation pattern of this deletion with respect to the classic aniridia phenotype. In other words, we caution against the over-interpretation of this finding as an example of a recessive form of aniridia at this time. It is hoped that our ongoing analysis of more classic aniridia cases in our highly consanguineous population will address this possibility. Future studies with even higher resolution microarrays are needed to further investigate the potential role of genomic copy number variation in classic aniridia patients without *PAX6* mutations.
